# Impact of insufficient sleep on dysregulated blood glucose control under standardised meal conditions

**DOI:** 10.1007/s00125-021-05608-y

**Published:** 2021-11-30

**Authors:** Neli Tsereteli, Raphael Vallat, Juan Fernandez-Tajes, Linda M. Delahanty, Jose M. Ordovas, David A. Drew, Ana M. Valdes, Nicola Segata, Andrew T. Chan, Jonathan Wolf, Sarah E. Berry, Matthew P. Walker, Timothy D. Spector, Paul W. Franks

**Affiliations:** 1 Genetic & Molecular Epidemiology Unit, Lund University Diabetes Centre, Department of Clinical Sciences, Lund University, Malmö, Sweden; 2 Center for Human Sleep Science, Department of Psychology, University of California, Berkeley, CA USA; 3 Diabetes Center, Department of Medicine, Massachusetts General Hospital and Harvard Medical School, Boston, MA USA; 4 JM-USDA-Human Nutrition Research Diabetes Center on Aging at Tufts University, Boston, MA USA; 5 IMDEA-Food, Madrid, Spain; 6 Clinical and Translational Epidemiology Unit, Massachusetts General Hospital and Harvard Medical School, Boston, MA USA; 7 Division of Gastroenterology, Massachusetts General Hospital and Harvard Medical School, Boston, MA USA; 8 NIHR Nottingham BRC at the Nottingham University Hospitals NHS Trust and University of Nottingham, Nottingham, UK; 9 Department of Cellular, Computational and Integrative Biology, University of Trento, Trento, Italy; 10 European Institute of Oncology Scientific Institute for Research, Hospitalization and Healthcare, Milan, Italy; 11 Zoe Ltd, London, UK; 12 Department of Nutritional Research, Kings College London, London, UK; 13 Department of Twins Research, Kings College London, London, UK; 14 Harvard Chan School of Public Health, Boston, MA USA

**Keywords:** Diet, Metabolic health, Person-centring, Postprandial glucose, Sleep

## Abstract

**Aims/hypothesis:**

Sleep, diet and exercise are fundamental to metabolic homeostasis. In this secondary analysis of a repeated measures, nutritional intervention study, we tested whether an individual’s sleep quality, duration and timing impact glycaemic response to a breakfast meal the following morning.

**Methods:**

Healthy adults’ data (*N* = 953 [41% twins]) were analysed from the PREDICT dietary intervention trial. Participants consumed isoenergetic standardised meals over 2 weeks in the clinic and at home. Actigraphy was used to assess sleep variables (duration, efficiency, timing) and continuous glucose monitors were used to measure glycaemic variation (>8000 meals).

**Results:**

Sleep variables were significantly associated with postprandial glycaemic control (2 h incremental AUC), at both between- and within-person levels. Sleep period time interacted with meal type, with a smaller effect of poor sleep on postprandial blood glucose levels when high-carbohydrate (low fat/protein) (*p*_*interaction*_ = 0.02) and high-fat (*p*_*interaction*_ = 0.03) breakfasts were consumed compared with a reference 75 g OGTT. Within-person sleep period time had a similar interaction (high carbohydrate: *p*_*interaction*_ = 0.001, high fat: *p*_*interaction*_ = 0.02). Within- and between-person sleep efficiency were significantly associated with lower postprandial blood glucose levels irrespective of meal type (both *p* < 0.03). Later sleep midpoint (time deviation from midnight) was found to be significantly associated with higher postprandial glucose, in both between-person and within-person comparisons (*p* = 0.035 and *p* = 0.051, respectively).

**Conclusions/interpretation:**

Poor sleep efficiency and later bedtime routines are associated with more pronounced postprandial glycaemic responses to breakfast the following morning. A person’s deviation from their usual sleep pattern was also associated with poorer postprandial glycaemic control. These findings underscore sleep as a modifiable, non-pharmacological therapeutic target for the optimal regulation of human metabolic health.

**Trial registration** ClinicalTrials.gov NCT03479866.

**Graphical abstract:**

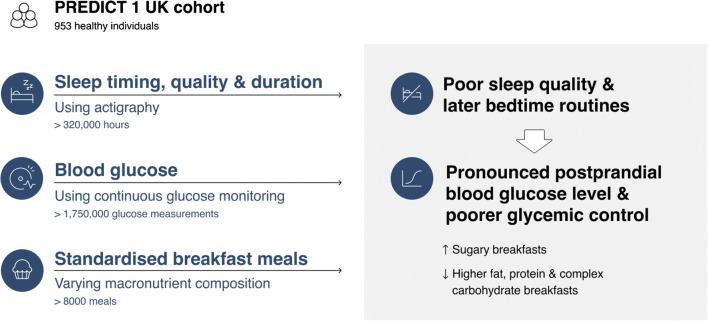

**Supplementary Information:**

The online version contains peer-reviewed but unedited supplementary material available at 10.1007/s00125-021-05608-y.

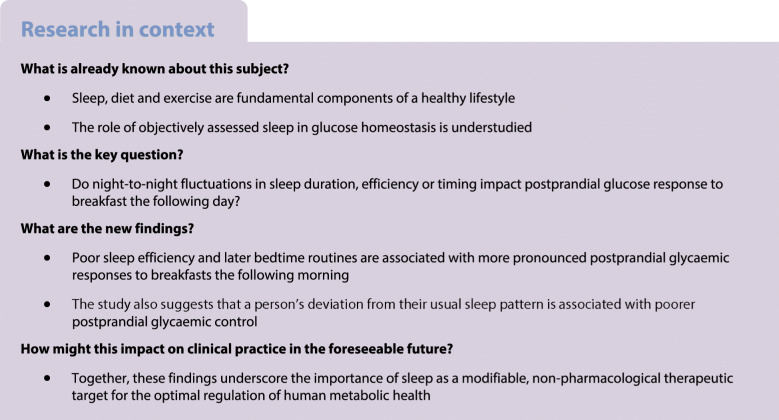



## Introduction

Diet, physical activity and sleep are increasingly recognised as core modifiable components of a healthy lifestyle. Sleep disorders often coalesce with other health ailments, and in this way provide a barometer of general health [1]. Sleep has direct causal effects on many life-threatening diseases such as CVD, obesity and type 2 diabetes [2–5]. Its disturbance (such as obstructive sleep apnoea) is associated with type 2 diabetes prevalence and complications [6] and can disrupt glucose homeostasis [7]. These and other data [8–10] point to a strong link between sleep quality/duration and glucose homeostasis. However, while there have been numerous large prospective cohort studies focused on the relationship between self-reported sleep, disease and wellbeing, objective data on sleep and postprandial glucose metabolism typically emanates from small studies conducted in tightly controlled settings and in specific population subgroups such as those suffering sleep disturbances owing to pregnancy, sleep apnoea, depression, obesity or diabetes [11]. Thus, the evidence base for potential recommendations concerning the effects of sleep on glucose metabolism in generally healthy people has considerable scope for expansion.

The purpose of this study was to investigate the relationship between sleep (duration, efficiency and midpoint) and postprandial glycaemic response to breakfasts of varying macronutrient composition in healthy adults from the UK and USA. Given the established role of sleep in glucose control in people with type 2 diabetes [12], we hypothesised that shorter sleep duration and poorer quality sleep would be associated with higher 2 h postprandial glucose incremental AUC (iAUC). We also hypothesised that there would be an interaction between sleep and meal type, meaning that the effect of sleep on postprandial glycaemic control would be modified by the macronutrient composition of a meal. We further investigated whether within-person sleep changes (deviations from the usual sleep pattern for a given participant) predicted that individual’s postprandial glycaemic control.

## Methods

### Participants

Participants from the UK and the USA were enrolled into the Personalized REsponses to DIetary Composition Trial 1 (PREDICT1; ClinicalTrials.gov registration no. NCT03479866), a single-arm, multiple-test-meal challenge study conducted over 14 consecutive days. Metabolic responses to various foods differing in macronutrient and energy content were determined in relation to each participant’s meal timing and sleep, as well as a range of biological characteristics [13]. The study was conducted between June 2018 and May 2019 and included 1002 generally healthy participants aged 18–65 years in the UK, as well as 100 generally healthy participants in the USA, with data being combined as there were no differences of effects for the two locations. Some of the UK participants were recruited from the TwinsUK research cohort, which includes both monozygotic twin (sharing the vast majority of their DNA sequence) and dizygotic twins (sharing roughly half of their DNA sequence) [14]. ‘Healthy’ was defined as being free of diagnosed diseases from the exclusion criteria. Exclusion criteria were as follows: ongoing inflammatory disease; cancer in the last 3 years (excluding skin cancer); long-term gastrointestinal disorders; taking immunosuppressants; using proton-pump inhibitors; diabetes; depression; eating disorder; and pregnancy. A full description of the PREDICT1 study protocol, including the rationale for the sample size, can be found elsewhere [13]. The current report describes secondary analyses of the PREDICT1 trial.

### Ethical approval

Ethical approval for the study was obtained from the Research Ethics Committee and Integrated Research Application System (IRAS 236407) and from the Institutional Review Board (Partners Healthcare IRB 2018P002078) in the UK and USA, respectively. Written informed consent was obtained from each participant immediately prior to enrolment in the trial during the baseline clinic visit.

### Test meal challenges

**Fig. 1:**
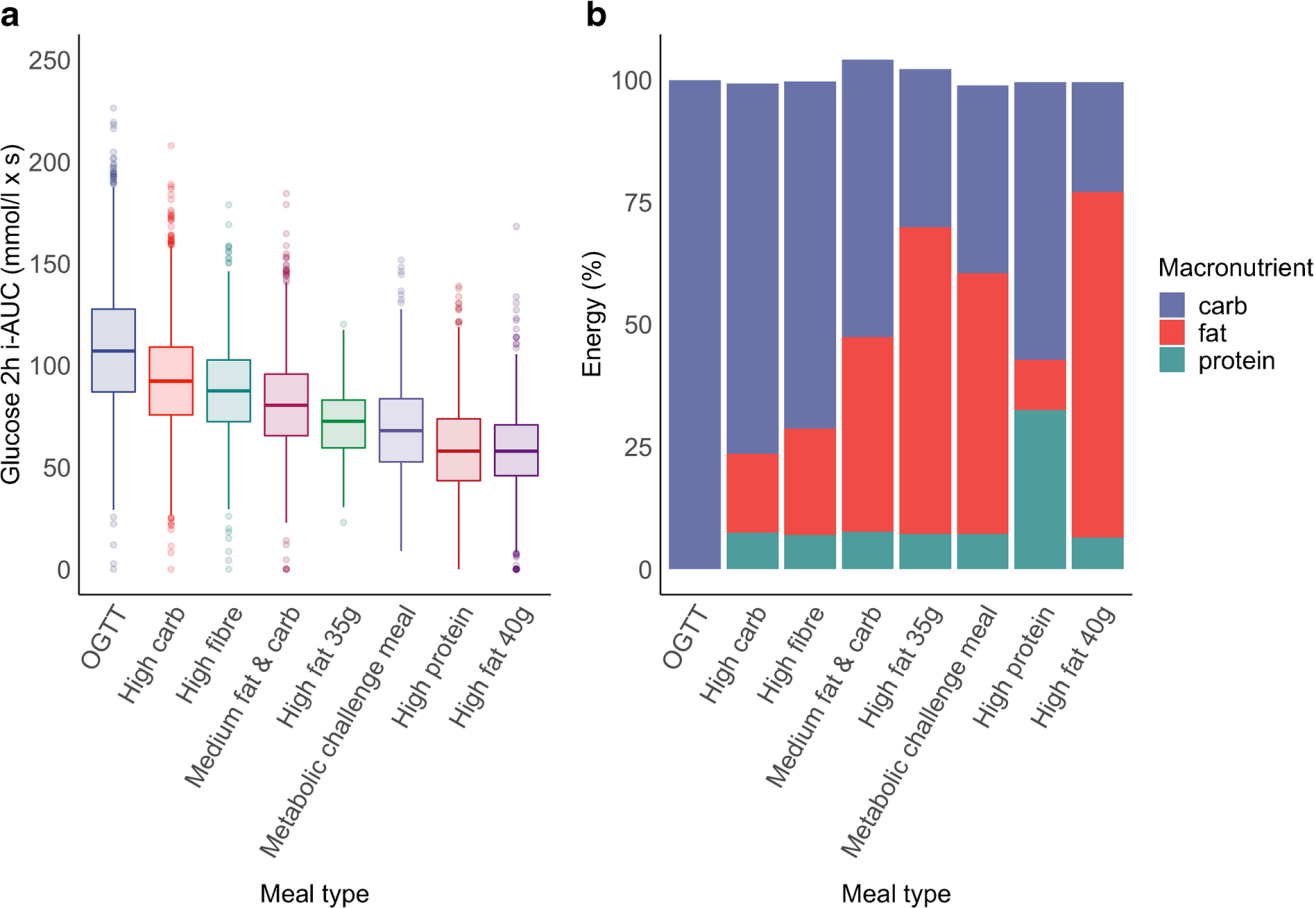
(**a**) Glucose 2 h iAUC distribution by test meal type. Box plot shows median and IQR; whiskers represent IQR and outliers (>1.5 × IQR) are shown. (**b**) Nutritional composition by test meal type. The iAUC data has also been published in [26]

Participants consumed standardised test meals of different nutritional composition (carbohydrates, fat, protein and dietary fibre). The meals were consumed either for breakfast or lunch in a randomised meal order and consisted of eight different standardised meals designated as follows: (1) metabolic challenge meal; (2) medium fat and carb; (3) high fat 35 g; (4) high carb (with low fat and protein); (5) 75 g OGTT, consisting of carbohydrates only; (6) high fibre; (7) high fat 40 g; and (8) high protein. The detailed nutritional composition of the test meals can be found in Fig. 1 (see also [13]).

Participants were asked to consume only their standardised breakfasts after no less than 8 h of fasting and to drink only still water during the fasting period. In addition, participants were requested to consume all their meals within 10 min, with the exception of the OGTT, which was to be consumed within 5 min. Participants were also requested to limit physical exercise during the 3 h period following the meal, as well as during the 8 h fasting period prior to its consumption. More detailed explanation of the test meal challenge protocol can be found elsewhere [13].

In this analysis, we included only the logged meals that complied with the protocol and passed logging accuracy assessment conducted by the study staff [13]. For example, overnight fasting was manually checked for each user, with the accuracy of meal being marked down if the time between last logged food the day before and the first on the following day was less than 8 h. Since glucose tolerance differs across the day owing to circadian effects that are independent of sleep [15], we excluded all meals except those consumed at breakfast from the analyses, thereby reducing non-sleep-related circadian effects on glucose regulation [15–17]. The OGTT was used as the reference meal type in the linear mixed-effects models, as the OGTT is the reference standard for assessing post-load glycaemic control in clinical settings.

### Sleep assessment

Activity and sleep were monitored using a wearable device with a triaxial accelerometer (AX3, Axivity; Newcastle Upon Tyne, UK). Accelerometers were fitted by trained research staff on the non-dominant wrist and were worn by the participants for the duration of the entire study.

Raw accelerometer data were analysed using GGIR, a multi-day raw accelerometer data analysis package in R [18, 19]. The raw accelerometer data from a wrist actigraphy units were converted into a single activity time series (BFEN) aggregated into epochs of 30 s. Sleep/wake detection was performed using the validated angle-z method implemented in GGIR [19]. Based on prior evidence linking sleep with metabolic system regulation [12], primary a priori target variables were as follows: (1) sleep duration or total sleep period time (SPT); (2) sleep efficiency (SE), where SE represents the ratio of time asleep to the total SPT; and (3) sleep midpoint, or the middle time point between bedtime and waking up (expressed in hours as a deviation from midnight). Sleep predictors were further decomposed into within-person effects (as opposed to between-person effects) using person-mean centring [20]. In the context of this study, such person-mean centring translated into the person’s 2 week average and his/her deviation from that average on a particular day.

A set of filtering conditions consistent with the practices of typical sleep analysis was applied to remove entries with invalid sleep data [21]. Nights with <2 h or >15 h of sleep or with SE <20% were excluded from the analysis, as they likely resulted from artefacts or poor-quality data (355 nights [2.5%]). Nights with more than 10% data classified as invalid were also excluded (399 nights [2.8%]). In addition, nights with a sleep onset during 08:00–17:00 h or a sleep offset after 00:00 h were excluded (26 nights [0.2%]). Finally, data from participants with less than 7 days of data and/or a percentage of invalid nights >35% (*n* = 89) were removed. We also removed data from participants who travelled in different time zones during the study, owing to disruption of their regular sleep patterns.

### Glucose assessment

Postprandial blood glucose was computed using a continuous glucose monitoring (CGM) wearable device (Freestyle Libre Pro; Abbott, Abbott Park, IL, USA), which measures interstitial glucose every 15 min. Monitors were fitted by trained research staff to the participant’s upper non-dominant arm and were worn for the entire study duration. Owing to the CGM’s calibration requirements, CGM data collected 12 h after fitting the device to a participant was used for analysis [13].

Incremental area under the blood glucose curve has been shown to accurately describe glycaemic responses to foods [22]. Accordingly, the primary outcome for this analysis was 2 h iAUC for postprandial glucose with interpolated baseline (glucose_iAUC0-2h_). The baseline value was interpolated at the meal start time based on the nearest CGM readings immediately before and after that time. The distribution of glucose_iAUC0-2h_ was right skewed. Thus, these data were transformed using a square-root transformation, which yielded better normalisation than conventional logarithmic transformations (electronic supplementary material [ESM] Fig. 1).

### Statistical analysis

Data analysis was performed using R version 3.6.1 [23]. To account for the individual uniqueness/variation of postprandial glycaemic responses (inter-individual variability) and the covariance structure resulting from repeated measurements (which would violate the independence of observations assumption necessary for linear regression), data were modelled using a linear mixed-effects model approach. We analysed data using lme4 package with lme4’s default REML (restricted or ‘residual’ maximum likelihood) criterion to estimate variance components [24]. The lmerTest package was used to calculate *p* values [25]; *p* < 0.05 was considered statistically significant.

All models were initially constructed with only the marginal terms for sleep and meal; the multiplicative interaction term for sleep × meal was added to the subsequent models. Similarly, all between-person models were subsequently re-run using the within-person approach. The outcome was glucose_iAUC0-2h_. Potential confounders were also taken into consideration. The models consisted of nine fixed effects: (1) different between-person and within-person sleep variables (SE, SPT, sleep midpoint); (2) standardised meal type; (3) sex; (4) age; (5) BMI; (6) zygosity (not a twin, monozygotic, dizygotic); (7) weekend; and (8) season; and (9) when applicable, sleep variable and standardised meal type interaction term to assess the combined effect of sleep variable and meal type on postprandial glucose metabolism. For random effects terms, we used participant ID (randomly generated study identification [ID] number) and family ID to allow for person-specific linear regressions, where participants have their own intercepts for the association of interest. Participant ID was nested within family ID to account for the data’s nested/hierarchical structure and the fact that measures coming from twins belonging to the same family might be more similar to each other owing to genetic and/or environmental factors. The models had random intercepts but no random slopes. Visual inspection of diagnostic plots did not reveal any strong deviations from homoscedasticity or normality, and all models were checked for multicollinearity. The main hypotheses tested were as follows: (1) whether the relationship between sleep (quality, timing or duration) and blood glucose is statistically different from the null hypothesis of no effect; and (2) whether the relationship between sleep and postprandial glycaemic response differs in magnitude conditional on the type of breakfast consumed. ESM Methods provides R codes for each of the main hypothesis tests. Owing to the hypothesis-driven nature of the analyses reported here, we considered a nominal *p* value threshold of 0.05 to be of statistical significance.

## Results

### Participant characteristics

Data from 953 participants were used to analyse 8395 sleep and postprandial responses (Fig. 1, Table 1). Consort diagrams for the PREDICT1 study can be found elsewhere [26].

**Table 1:** Participant characteristics

Characteristic	PREDICT1 UK (*n* = 869)	PREDICT1 USA (*n* = 84)	Overall (*n* = 953)
Sex, *n* (%)	636 (73.2)	55 (65.5)	691 (72.5)
Female	233 (26.8)	29 (34.5)	262 (27.5)
Male			
Age, years	46.2 ± 11.9	41.8 ± 12.8	45.8 ± 12.0
Zygosity, *n* (%)			
NT	303 (34.9)	84 (100)	387 (40.6)
DZ	151 (17.4)	0 (0)	151 (15.8)
MZ	415 (47.8)	0 (0)	415 (43.5)
BMI, kg/m^2^	25.7 ± 5.01	25.7 ± 4.26	25.7 ± 4.95
SE, %^a^	0.89 ± 0.057	0.89 ± 0.062	0.89 ± 0.058
SPT, h^a^	7.69 (6.87, 8.48)	7.64 (6.66, 8.61)	7.68 (6.86, 8.50)
Sleep midpoint as a deviation from midnight, h^a^	3.25 ± 1.15	2.87 ± 1.30	3.21 ± 1.17
Glucose 2 h iAUC, mmol/l × s^a^	83.0 ± 31.3	81.5 ± 32.6	82.8 ± 31.4
Meal type, *n* (%)^a^			
OGTT	1507 (19.8)	136 (17.2)	1643 (19.6)
High carb	1475 (19.4)	140 (17.7)	1615 (19.2)
High fat 35 g	215 (2.8)	0 (0)	215 (2.6)
High fat 40 g	1105 (14.5)	144 (18.2)	1249 (14.9)
High fibre	683 (9.0)	76 (9.6)	759 (9.0)
High protein	844 (11.1)	75 (9.5)	919 (10.9)
MCM	319 (4.2)	71 (9.0)	390 (4.6)
Medium fat and carb	1457 (19.2)	148 (18.7)	1605 (19.1)

Values are mean ± SD, median (Q1, Q3)

^a^meals *n* = 7605 for PREDICT1 UK, *n* = 790 for PREDICT1 USA and *n* = 8395 overall)

DZ, dizygotic twin; MCM, metabolic challenge meal; MZ, monozygotic twin; NT, not a twin

To provide insights into the effects of sleep on postprandial glycaemic control at both within-individual and group level, we present these two sets of results separately. All models were adjusted for age, sex, BMI, meal type, zygosity, weekend and season. We found no significant interactions between postprandial glucose and sex, meaning that there was no evidence to believe the results are different for the two sexes.

### SPT

#### Between-participant effects

**Fig. 2:**
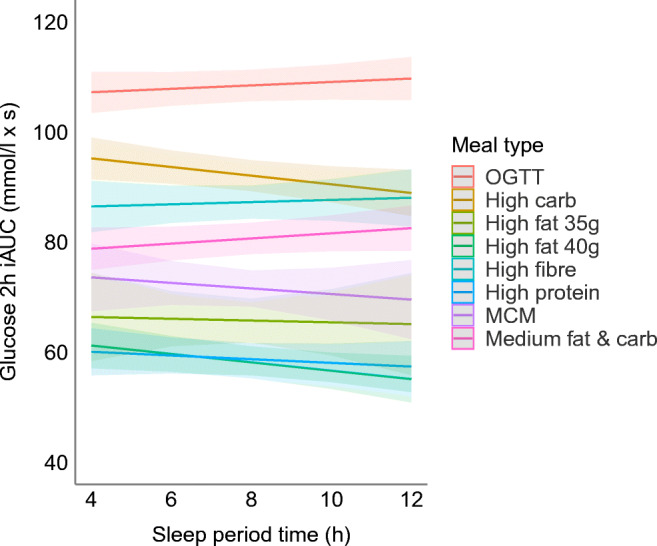
Meal type and SPT interaction effects on glucose 2 h iAUC. The plot fits the model using the marginal effects of the interaction terms with the standard errors. MCM, metabolic challenge meal

We found no statistically significant association between SPT and glucose_iAUC0-2h_ (ESM Table 1). However, we found a statistically significant interaction between SPT and meal type, with SPT having a significantly negative association with glucose_iAUC0-2h_ following high-carbohydrate and high-fat breakfasts (β_SPT × high carb_ = −1.10, *p*_SPT × high carb_ = 0.021; β_SPT × high fat 40g_ = −1.07, *p*_SPT × high fat 40g_ = 0.031) (Fig. 2 and ESM Table 1).

#### Within-participant effects

The person-centred model revealed an effect similar to that of the between-person model, suggesting that an SPT that exceeded a person’s average SPT is associated with lower postprandial response following a high-carbohydrate or high-fat breakfast (β_SPT-person × high carb_ = −1.84, *p*_SPT-person × high carb_ = 0.001; β_SPT-person × high fat 40g_ = −1.36, *p*_SPT-person × high fat 40g_ = 0.019) (ESM Table 2).

### SE

#### Between-participant effects

A larger between-person SE was significantly associated with lower glucose_iAUC0-2h_ (β_SE_ = −10.48 [95% CI −19.85, −1.11], *p*_SE_ = 0.028) (ESM Table 3). In a model assessing the interaction of SE and meal type, higher SE was still significantly associated with lower glucose_iAUC0-2h_ (β_SE_ = −19.18 [95% CI −36.46, −1.91], *p*_SE_ = 0.030), but none of the interaction terms was statistically significant (ESM Table 3). This association remained similar after additional adjustment for SPT (*p* = 0.016).

#### Within-participant effects

A greater within-person SE was significantly associated with lower glucose_iAUC0-2h_ (β_SE_ = −11.93 [95% CI_SE_ −21.83, −2.04], *p*_SE_ = 0.018), meaning that achieving better than one’s average SE was associated with better postprandial glycaemic control the following day (ESM Table 4). In the model with SE and meal type interaction term, higher within-person SE was still significantly associated with lower glucose_iAUC0-2h_ (β_SE_ = −29.75 [95% CI_SE_ −52.29, −7.21], *p*_SE_ = 0.010) (ESM Table 4).

### Sleep midpoint

#### Between-participant and within-participant effects

A later between-person sleep midpoint (expressed in hours as a deviation from midnight) was significantly associated with higher glucose iAUC (β_midpoint_ = 0.52 [95% CI_midpoint_ 0.04, 1.01], *p*_midpoint_ = 0.035) (Table 2). This effect was largely driven by sleep onset (going to bed later) rather than sleep offset (waking up later) (ESM Tables 5–7). The effect ceased to be significant in a model incorporating an interaction term between sleep midpoint and meal type (Table 2). In the within-person effects model, having a later sleep midpoint than one’s average had a similar coefficient (β_midpoint_ = 0.51 [95% CI_midpoint_ 0.00, 1.02], *p*_midpoint_ = 0.051).

**Table 2:** Between-person sleep midpoint and postprandial blood glucose concentrations

Predictor	Model without interaction			Model with interaction		
	Estimate	95% CI	*p* value	Estimate	95% CI	*p* value
(Intercept)	81.13	72.61, 89.65	<0.001	80.70	71.90, 89.51	<0.001
Sleep midpoint^a^	0.52	0.04, 1.01	0.035	0.69	−0.17, 1.55	0.116
Meal (high carb)	−15.92	−17.23, −14.60	<0.001	−14.83	−18.74, −10.93	<0.001
Meal (high fat 35 g)	−42.42	−45.38, −39.46	<0.001	−41.66	−50.39, −32.93	<0.001
Meal (high fat 40 g)	−49.98	−51.39, −48.58	<0.001	−48.17	−52.31, −44.03	<0.001
Meal (high fibre)	−21.20	−22.82, −19.57	<0.001	−17.62	−22.41, −12.83	<0.001
Meal (high protein)	−49.56	−51.11, −48.01	<0.001	−51.21	−55.71, −46.71	<0.001
Meal (MCM at home)	−36.55	−38.67, −34.43	<0.001	−33.10	−39.73, −26.46	<0.001
Meal (medium fat and carb)	−27.90	−29.19, −26.60	<0.001	−29.06	−32.94, −25.18	<0.001
Sleep midpoint × Meal (high carb)				−0.33	−1.46, 0.80	0.565
Sleep midpoint × Meal (high fat 35 g)				−0.23	−2.77, 2.31	0.860
Sleep midpoint × Meal (high fat 40 g)				−0.58	−1.82, 0.66	0.358
Sleep midpoint × Meal (high fibre)				−1.09	−2.46, 0.29	0.121
Sleep midpoint × Meal (high protein)				0.49	−0.79, 1.77	0.455
Sleep midpoint × Meal (MCM at home)				−1.13	−3.19, 0.92	0.280
Sleep midpoint ×				0.37	−0.77, 1.51	0.525

Random effects terms for both models: intraclass correlation coefficient, 0.45; *N* = 953_username_; *N* = 765_family_id_; *n* observations 8395; marginal *R*^2^/conditional *R*^2^ = 0.363/0.652

^a^Sleep midpoint is expressed in hours as a deviation from midnight

MCM, Metabolic challenge meal

## Discussion

Here, we describe for the first time how sleep duration, quality and midpoint associate with postprandial glucose metabolism in healthy individuals. While sleep is generally recognised as one of the pillars of good health, the data reported here suggest that one-size-fits-all sleep recommendations are suboptimal, particularly in the context of postprandial glycaemic control, a key component of diabetes prevention.

By analysing both between-person and within-person effects, this study provides unique and powerful insights into both population-level and person-level effects of sleep on metabolic health. Notably, our data suggest that sleep duration, efficiency and midpoint are important determinants of postprandial glycaemic control at a population level, while illustrating that to optimise sleep recommendations it is likely necessary to tailor these to the individual.

Diet, sleep and health are interrelated. Several studies have investigated the relationship between sleep duration and glucose metabolism in pregnant women and reported a positive association between reduced sleep duration and impaired glucose metabolism [4, 27]. However, we are not aware of any other studies to date that investigate the relationship between objectively assessed sleep characteristics and postprandial glucose metabolism in generally healthy adults. The findings from this intervention study, with repeated test meal challenges, combined with objective assessments of sleep and blood glucose from a large population, complement a relatively small body of knowledge around a topic that is likely to be of high relevance for diabetes prevention [26]. Importantly, many earlier studies were undertaken in sleep laboratories with small sample size [11]. While the controlled environment of such studies is necessary to understand specific aspects of sleep and metabolism, the real-world, community-dwelling setting of the current study provides novel insights into how habitual sleep affects metabolic health.

The main analyses in this study focused on interactions of sleep and meal type and selected the OGTT as the ‘breakfast’ against which all other breakfast meals were compared. This is primarily because the OGTT is the standard clinical test used to assess glucose tolerance. While this is not a realistic breakfast meal, there is a growing trend, particularly among younger people [28], to consume energy drinks as a pick-me-up the morning after a poor night’s sleep, with the sugar content of a 75 g OGTT equating to roughly two to three servings of standard energy drinks.

### SPT

With SPT being a marker of sleep duration, the lack of a significant marginal effect in the model without interactions indicates that sleep duration is not a major determinant of glucose metabolism. While this finding does not support some prior studies that have demonstrated a potential link between decreased sleep duration and insulin resistance, it is consistent with findings from a randomised controlled trial of 42 normal-weight adult short sleepers [11]. This may be because the effect of sleep duration in glycaemic control may be non-linear, with sleep affecting glucose metabolism only once sleep duration dips below a specific bound [11]. Moreover, sleep duration for the vast majority of PREDICT1 participants fell within the recommended range, as indicated by the mean 6.87 h of sleep within the lowest quartile of the SPT distribution (Table 1). Accordingly, this study may have been underpowered to detect an association between sleep duration and postprandial glycaemic control.

We found a significant statistical interaction between SPT and meal type, with high-carbohydrate meals and high-fat meals resulting in significantly lower glucose iAUCs compared with the OGTT reference, which contained only sugar. Although this study did not include pregnant women, the interaction effect between carbohydrate-rich meals and SPT is consistent with the results reported in a prior study in which reduced sleep was associated with impaired carbohydrate metabolism in pregnant women [29]. Thus, we conclude that the SPT has a similar impact on postprandial carbohydrate metabolism in men and pregnant and non-pregnant women. Additionally, the significant interaction between SPT and high-fat meals is supported by the finding that sleep disruption in fat-fed mice negatively affected glucose metabolism, with metabolism improving after recovery sleep [30]. As for the within-person SPT model, our findings suggest that both a longer SPT in general, as well as having a longer SPT than one is used to, are associated with improved postprandial glycaemic control the following morning. The presence of a significant finding with the SPT × high-carbohydrate meal interaction term in the person-centred model suggests that getting more sleep than usual might be more important for postprandial glycaemic control than the absolute amount of sleep achieved. This insight offers a potential avenue for personalised (within-person) sleep interventions.

### SE

Better SE, between-person as well as within-person, was significantly associated with lower glucose iAUC, meaning that better SE, which is a proxy of sleep quality, was associated with better glucose management following breakfast. However, the absence of SE × meal interactions suggests that SE may be beneficial for postprandial glucose response irrespective of meal composition. Although there is not much research on SE and glucose metabolism in healthy adults, our findings concur with a recent meta-analysis in which poor sleep quality was associated with poor glycaemic control in individuals with type 2 diabetes [12]. Moreover, since SE can be viewed as a proxy for sleep quality, and because we found a significant association between SE and glucose regulation, but not between SPT and glucose regulation, our findings suggest that sleep quality is more important than sleep duration with respect to glycaemic control. However, sleep apnoea is known to affect SE, and sleep apnoea was not measured in the PREDICT1 study.

### Sleep midpoint

The presence of significant effects in both between-person and within-person sleep midpoint models adjusted for sleep duration suggests a novel finding that later sleep midpoint, such as that caused by going to bed later, is associated with impaired postprandial glucose response to breakfast the following morning. This concurs with the proposition that human metabolic health is determined to a considerable extent by chronobiology [31]. Alternatively, later sleep midpoint may reflect alteration of sleep stages caused by going to bed later. Thus, the significance of later sleep midpoint may also be indicative of the role of specific sleep stages, such as slow-wave sleep, on glucose metabolism, supporting the view that treating slow-wave sleep disorders may help improve glycaemic control [32].

The 2 h glucose iAUC response to an OGTT breakfast is roughly twice that following a high-fat breakfast, indicating that a high-fat breakfast might help to mitigate the detrimental effects of poor sleep on postprandial glycaemia. Although comparing areas may have its drawbacks, for those whose sleep is often compromised, these effects may be cumulative. Thus, over time, there may be a meaningful clinical impact on glycaemic health. Nevertheless, because of the short duration of the current study, we are not able to assess this hypothesis.

Much of the research linking poor sleep with altered glucose metabolism is based upon observational studies, meaning that the pathophysiological mechanisms behind the associations reported here are not well understood [33]. However, poor sleep quality (measured by sleep fragmentation in healthy volunteers) appears to alter glucose responses through shifting sympathovagal balance and morning cortisol levels, which could in turn lead to decreased insulin sensitivity, increased hepatic glucogenesis and decreased insulin secretion [33]. In addition to cortisol levels, growth hormone, the secretion of which is sleep-dependent and which is essential for metabolic regulation, could also be at play [34–36]. Moreover, the glucose intolerance observed elsewhere in sleep-deprived individuals may derive from dysregulation of sympathetic and parasympathetic control of pancreatic function [37].

### Strengths and limitations

This study significantly extends our understanding of the interplay between sleep and metabolic health. First, the fairly limited literature on sleep and postprandial blood glucose regulation is dominated by small studies that focus on populations with comorbid conditions (e.g. diabetes and obstructive sleep apnoea). In the few larger published studies, sleep has typically been assessed through self-report, which may be prone to bias. Moreover, most studies are cross-sectional and based in highly controlled environments. By contrast, our study is set within a large prospective cohort of generally healthy individuals, in whom high-resolution objectively assessed time-series sleep and glucose data were obtained. These design features made it possible to look at both intra- and inter-individual variation during the analyses, have generalisable results and shed light on cause and effect. The risk of non-compliance due to a non-clinical setting was addressed by high levels of staff support and all data points were checked for compliance and validity. We also examined the effects of within-person sleep measures, thus broadening the scope of previous studies that up to now only included between-person differences. In addition, instead of relying on fasting blood assays, the study focused on postprandial glucose, which is more relevant to everyday life scenarios because most people find themselves most often in a postprandial state during waking hours [26].

Notwithstanding the strengths of the study, it is limited in that no screening was performed for sleep disorders (e.g. sleep apnoea and insomnia), meaning that we could not control for disorders that have been shown to be associated with impaired glucose tolerance [38, 39]. In addition, while actigraphy overcomes many limitations of self-report measures, it is not as accurate as polysomnography in estimating sleep duration and efficiency and does not offer insight into individual sleep stages. The distribution of meal types was imbalanced, with high-fat and high-carbohydrate standardised meals having the highest number of entries. Thus, it is possible that analyses focused on the non-high-carbohydrate and non-high-fat meals may have lacked statistical power. An additional limitation is that owing to the free-living nature of the trial, physical activity levels varied within and between individuals, which may have interacted with sleep and meal type to affect blood glucose concentrations, a hypothesis that our study was not powered to examine.

Future studies assessing the impact of sleep stages on postprandial blood glucose levels are likely to extend the findings of the current analysis, as would exploration of these effects in individuals who are sleep-deprived owing to shift work or endogenous sleep disorders such as sleep apnoea.

### Conclusion

Overall, this study suggests that sleep duration, quality and midpoint are important modifiable lifestyle features for improving postprandial glucose metabolism in healthy adults. As a consequence, this study’s findings may inform lifestyle strategies to improve postprandial blood glucose levels, focusing on earlier bedtime routines and maximising high-quality uninterrupted sleep. A combination of both generalised and more personalised sleep guidelines is likely required to ensure optimal metabolic health per se and maximise the effectiveness of guidelines for diabetes prevention.

## Supplementary Information

ESM (PDF 120 kb)

## Data Availability

The data from the baseline clinic visit of PREDICT1 belong to the department of Twin Research at King’s College London. Information about the data access application process can be found at: https://twinsuk.ac.uk/resources-for-researchers/access-our-data/. The data from the at-home phase of the trial, which belong to Zoe Ltd. and were used under license, are not publicly available. However, the data can be made available granted a reasonable request and a permission from Zoe Ltd.

## Abbreviations

CGM: Continuous glucose monitor
iAUC: Incremental AUC
PREDICT: Personalized REsponses to DIetary Composition Trial
SE: Sleep efficiency
SPT: Sleep period time
