# Supplementary material for: Impact of insufficient sleep on dysregulated blood glucose control under standardised meal conditions
Source: Diabetologia. 2021 Nov 30;65(2):356–65. doi: 10.1007/s00125-021-05608-y (PMC8741723; doi:10.1007/s00125-021-05608-y)
Supplement: Supplementary file 1 — (PDF 120 kb) [file 125_2021_5608_MOESM1_ESM.pdf]

Extra Supplementary Materials for *Tsereteli et al*.

ESM Methods

Supplementary R codes for linear mixed-model hypothesis tests (marginal terms are noted in interaction models for the sake of clarity, although R does not require these to be explicitly stated within models)

```
####### SPT regular #######
model_spt <- lmer(meal_iauc_sqrt ~ spt + meal + sex + age + bmi +
zygosity + weekend + season + (1|family_id/username), data = data)

# With SPT x meal interaction term
model_spt_int <- lmer(meal_iauc_sqrt ~ spt×Meal + spt + meal + sex +
age + bmi + zygosity + weekend + season + (1|family_id/username), data
= data)

####### SPT person-centered #######
model_spt_person <- lmer(meal_iauc_sqrt ~ spt_personcentered + meal +
sex + age + bmi + zygosity + weekend + season + (1|family_id/username),
data = data)

With SPT-person x meal interaction term
model_spt_person_int <- lmer(meal_iauc_sqrt ~ spt_personcentered×Meal +
spt_personcentered + Meal + sex + age + bmi + zygosity + weekend +
season + (1|family_id/username), data = data)

####### SE regular #######
model_se <- lmer(meal_iauc_sqrt ~ se + meal + sex + age + bmi +
zygosity + weekend + season+ (1|family_id/username), data = data)

# With interaction term
model_se_int <- lmer(meal_iauc_sqrt ~ se×Meal + se + Meal + sex + age +
bmi + zygosity + weekend + season+ (1|family_id/username), data = data)

####### SE person-centered ########
model_se_person <- lmer(meal_iauc_sqrt ~ se_personcentered + meal + sex
+ age + bmi + zygosity + weekend + season + (1|family_id/username),
data = data)

# With interaction
model_se_person_int <- lmer(meal_iauc_sqrt ~ se_personcentered×Meal +
se_personcentered + Meal + sex + age + bmi + zygosity + weekend +
season + (1|family_id/username), data = data)


####### Sleep midpoint ########
model_times <- lmer(meal_iauc_sqrt ~ sleepmidpoint_hr + spt + meal +
sex + age + bmi + zygosity + weekend + season+ (1|family_id/username),
data = data)

# With interaction
model_times_int <- lmer(meal_iauc_sqrt ~ sleepmidpoint_hr×Meal +
sleepmidpoint_hr + Meal + spt + sex + age + bmi + zygosity + weekend +
season+ (1|family_id/username), data = data)
```

ESM Tables

Supplementary Table 1. Between-person sleep period time and postprandial glucose

	**Model without interaction**			**Model with interaction**		
Predictors	*Estimates*	*CI*	*p*	*Estimates*	*CI*	*p*
(Intercept)	81.95	73.74, 90.15	<0.001	78.44	69.18, 87.70	<0.001
SPT	-0.15	-0.48, 0.18	0.376	0.31	-0.33, 0.95	0.343
Meal [High Carb]	-15.97	-17.28, -14.66	<0.001	-7.62	-14.83, -0.42	0.038
Meal [High Fat 35g]	-42.54	-45.50, -39.58	<0.001	-38.94	-54.59, -23.30	<0.001
Meal [High Fat 40g]	-50.06	-51.46, -48.66	<0.001	-41.73	-49.42, -34.03	<0.001
Meal [High Fibre]	-21.21	-22.84, -19.58	<0.001	-20.30	-29.26, -11.34	<0.001
Meal [High Protein]	-49.55	-51.10, -48.00	<0.001	-44.57	-52.65, -36.49	<0.001
Meal [MCM at home]	-36.62	-38.74, -34.50	<0.001	-30.37	-42.51, -18.22	<0.001
Meal [Medium fat/carb]	-27.92	-29.22, -26.63	<0.001	-29.06	-36.18, -21.95	<0.001
SPT × Meal [High Carb]				-1.10	-2.03, -0.17	0.021
SPT × Meal [High Fat 35g]				-0.47	-2.49, 1.54	0.645
SPT × Meal [High Fat 40g]				-1.07	-2.05, -0.10	0.031
SPT × Meal [High Fibre]				-0.11	-1.27, 1.04	0.847
SPT × Meal [High Protein]				-0.65	-1.68, 0.39	0.221
SPT × Meal [MCM at home]				-0.82	-2.39, 0.76	0.309
SPT × Meal [Medium fat/carb]				0.16	-0.76, 1.07	0.737
Random Effects						
ICC	0.45			0.45		
N	953 $_{username}$			953 $_{username}$		
	765 $_{family\_id}$			765 $_{family\_id}$		
Observations (*n* meals)	8395			8395		
Marginal R^2 / Conditional R^2	0.363 / 0.651			0.363 / 0.652		

SPT, sleep period time; MCM, metabolic challenge meal

Supplementary Table 2. Within-person sleep period time and postprandial glucose

	Model without interaction			Model with interaction		
Predictors	*Estimates*	*CI*	*p*	*Estimates*	*CI*	*p*
(Intercept)	80.62	72.98, 88.26	<0.001	80.62	72.97, 88.26	<0.001
SPT-person	-0.15	-0.48, 0.19	0.396	0.70	-0.05, 1.46	0.069
Meal [High Carb]	-15.97	-17.28, -14.66	<0.001	-16.15	-17.47, -14.84	<0.001
Meal [High Fat 35g]	-42.54	-45.50, -39.58	<0.001	-42.57	-45.53, -39.62	<0.001
Meal [High Fat 40g]	-50.06	-51.47, -48.66	<0.001	-50.03	-51.43, -48.62	<0.001
Meal [High Fibre]	-21.21	-22.84, -19.58	<0.001	-21.18	-22.82, -19.55	<0.001
Meal [High Protein]	-49.55	-51.10, -48.00	<0.001	-49.57	-51.12, -48.01	<0.001
Meal [MCM at home]	-36.62	-38.74, -34.50	<0.001	-36.62	-38.75, -34.48	<0.001
Meal [Medium fat/carb]	-27.92	-29.22, -26.63	<0.001	-27.89	-29.19, -26.60	<0.001
SPT-person × Meal [High Carb]				-1.84	-2.96, -0.72	0.001
SPT-person × Meal [High Fat 35g]				-0.81	-3.28, 1.66	0.520
SPT-person × Meal [High Fat 40g]				-1.36	-2.49, -0.22	0.019
SPT-person × Meal [High Fibre]				-0.44	-1.81, 0.93	0.531
SPT-person × Meal [High Protein]				-0.98	-2.24, 0.27	0.123
SPT-person × Meal [MCM at home]				-0.82	-2.78, 1.15	0.415
SPT-person × Meal [Medium fat/carb]				-0.53	-1.64, 0.58	0.351
Random Effects						
ICC	0.45			0.45		
N	953 $_{username}$			953 $_{username}$		
	765 $_{family\_id}$			765 $_{family\_id}$		
Observations (*n* meals)	8395			8395		
Marginal R^2 / Conditional R^2	0.363 / 0.651			0.363 / 0.652		

SPT-person, person-centered sleep period time; MCM, metabolic challenge meal

Supplementary Table 3. Between-person sleep efficiency and postprandial glucose

	Model without interaction			Model with interaction		
Predictors	*Estimates*	*CI*	*p*	*Estimates*	*CI*	*p*
(Intercept)	90.95	79.81, 102.10	<0.001	98.68	81.61, 115.75	<0.001
SE	-10.48	-19.85, -1.11	0.028	-19.18	-36.46, -1.91	0.030
Meal [High Carb]	-15.92	-17.23, -14.61	<0.001	-25.93	-46.60, -5.26	0.014
Meal [High Fat 35g]	-42.50	-45.45, -39.54	<0.001	-65.39	-113.50, -17.28	0.008
Meal [High Fat 40g]	-49.99	-51.40, -48.59	<0.001	-69.55	-92.12, -46.97	<0.001
Meal [High Fibre]	-21.18	-22.81, -19.55	<0.001	-29.48	-55.23, -3.73	0.025
Meal [High Protein]	-49.51	-51.06, -47.96	<0.001	-61.18	-85.50, -36.86	<0.001
Meal [MCM at home]	-36.49	-38.61, -34.37	<0.001	-36.18	-70.31, -2.06	0.038
Meal [Medium fat/carb]	-27.88	-29.18, -26.59	<0.001	-30.94	-51.20, -10.67	0.003
SE × Meal [High Carb]				11.22	-11.90, 34.35	0.341
SE × Meal [High Fat 35g]				25.58	-28.06, 79.21	0.350
SE × Meal [High Fat 40g]				21.83	-3.32, 46.99	0.089
SE × Meal [High Fibre]				9.31	-19.51, 38.12	0.527
SE × Meal [High Protein]				13.10	-14.12, 40.31	0.346
SE × Meal [MCM at home]				-0.25	-38.13, 37.63	0.990
SE × Meal [Medium fat/carb]				3.43	-19.25, 26.11	0.767
Random Effects						
ICC	0.45			0.45		
N	953 username			953 username		
	765 family_id			765 family_id		
Observations (*n* meals)	8395			8395		
Marginal R^2 / Conditional R^2	0.363 / 0.652			0.363 / 0.651		

SE, sleep efficiency; MCM, metabolic challenge meal

Supplementary Table 4. Within-person sleep efficiency and postprandial glucose

	**Model without interaction**			**Model with interaction**		
Predictors	*Estimates*	*CI*	*p*	*Estimates*	*CI*	*p*
(Intercept)	81.83	74.16, 89.50	<0.001	81.92	74.25, 89.58	<0.001
SE-person	-11.93	-21.83, -2.04	0.018	-29.75	-52.29, -7.21	0.010
Meal [High Carb]	-15.92	-17.22, -14.61	<0.001	-15.90	-17.21, -14.59	<0.001
Meal [High Fat 35g]	-42.49	-45.45, -39.54	<0.001	-42.51	-45.47, -39.55	<0.001
Meal [High Fat 40g]	-49.98	-51.38, -48.57	<0.001	-50.07	-51.48, -48.65	<0.001
Meal [High Fibre]	-21.18	-22.81, -19.55	<0.001	-21.16	-22.79, -19.53	<0.001
Meal [High Protein]	-49.51	-51.06, -47.95	<0.001	-49.47	-51.02, -47.92	<0.001
Meal [MCM at home]	-36.48	-38.60, -34.36	<0.001	-36.35	-38.50, -34.20	<0.001
Meal [Medium fat/carb]	-27.88	-29.17, -26.59	<0.001	-27.86	-29.15, -26.56	<0.001
SE-person × Meal [High Carb]				9.17	-23.31, 41.66	0.580
SE-person × Meal [High Fat 35g]				27.38	-44.92, 99.67	0.458
SE-person × Meal [High Fat 40g]				36.81	1.32, 72.30	0.042
SE-person × Meal [High Fibre]				17.37	-23.12, 57.85	0.400
SE-person × Meal [High Protein]				48.23	10.38, 86.09	0.013
SE-person × Meal [MCM at home]				0.52	-56.04, 57.09	0.986
SE-person × Meal [Medium fat/carb]				16.14	-16.41, 48.70	0.331
Random Effects						
ICC	0.45			0.45		
N	953 $_{username}$			953 $_{username}$		
	765 $_{family\_id}$			765 $_{family\_id}$		
Observations (*n* meals)	8395			8395		
Marginal R^2 / Conditional R^2	0.363 / 0.651			0.364 / 0.651		

SE-person, person-centered sleep efficiency; MCM, metabolic challenge meal

	Model without interaction			Model with interaction		
Predictors	*Estimates*	*CI*	*p*	*Estimates*	*CI*	*p*
(Intercept)	81.09	72.57, 89.61	<0.001	80.97	72.44, 89.50	<0.001
Sleep onset[a]	0.53	0.05, 1.02	0.031	0.37	-0.41, 1.15	0.348
Meal [High Carb]	-15.92	-17.23, -14.60	<0.001	-15.70	-17.12, -14.28	<0.001
Meal [High Fat 35g]	-42.42	-45.38, -39.46	<0.001	-42.35	-45.55, -39.14	<0.001
Meal [High Fat 40g]	-49.98	-51.39, -48.58	<0.001	-49.84	-51.44, -48.23	<0.001
Meal [High Fibre]	-21.20	-22.83, -19.57	<0.001	-21.53	-23.28, -19.78	<0.001
Meal [High Protein]	-49.56	-51.11, -48.01	<0.001	-49.18	-50.83, -47.52	<0.001
Meal [MCM at home]	-36.55	-38.67, -34.43	<0.001	-36.82	-39.26, -34.37	<0.001
Meal [Medium fat/carb]	-27.90	-29.19, -26.60	<0.001	-27.76	-29.19, -26.34	<0.001
Sleep onset × Meal [High Carb]				0.39	-0.59, 1.36	0.437
Sleep onset × Meal [High Fat 35g]				0.11	-2.00, 2.22	0.918
Sleep onset × Meal [High Fat 40g]				0.21	-0.83, 1.25	0.689
Sleep onset × Meal [High Fibre]				-0.69	-1.87, 0.48	0.246
Sleep onset × Meal [High Protein]				0.77	-0.34, 1.88	0.173
Sleep onset × Meal [MCM at home]				-0.31	-1.98, 1.36	0.716
Sleep onset × Meal [Medium fat/carb]				0.22	-0.77, 1.20	0.664
Random Effects						
ICC	0.45			0.45		
N	953 $_{username}$			953 $_{username}$		
	765 $_{family\_id}$			765 $_{family\_id}$		
Observations (*n* meals)	8395			8395		
Marginal R^2 / Conditional R^2	0.363 / 0.652			0.363 / 0.652		

Supplementary table 5. Between-person sleep onset and postprandial glucose

[a] Sleep onset is expressed in hours as a deviation from midnight
MCM, metabolic challenge meal

Supplementary Table 6. Between-person sleep offset and postprandial glucose

	Model without interaction			Model with interaction		
Predictors	*Estimates*	*CI*	*p*	*Estimates*	*CI*	*p*
(Intercept)	81.19	72.67, 89.71	<0.001	78.67	69.22, 88.12	<0.001
Sleep offset [a]	0.51	0.02, 0.99	0.040	0.88	0.12, 1.64	0.024
Meal [High Carb]	-15.92	-17.23, -14.60	<0.001	-9.88	-16.78, -2.99	0.005
Meal [High Fat 35g]	-42.42	-45.38, -39.46	<0.001	-39.49	-55.17, -23.81	<0.001
Meal [High Fat 40g]	-49.99	-51.39, -48.58	<0.001	-42.67	-50.21, -35.14	<0.001
Meal [High Fibre]	-21.20	-22.82, -19.57	<0.001	-14.85	-23.44, -6.27	0.001
Meal [High Protein]	-49.56	-51.11, -48.00	<0.001	-49.47	-57.25, -41.69	<0.001
Meal [MCM at home]	-36.55	-38.67, -34.43	<0.001	-27.55	-39.94, -15.16	<0.001
Meal [Medium fat/carb]	-27.90	-29.19, -26.60	<0.001	-30.11	-36.91, -23.30	<0.001
Sleep offset × Meal [High Carb]				-0.86	-1.82, 0.10	0.081
Sleep offset × Meal [High Fat 35g]				-0.41	-2.60, 1.77	0.710
Sleep offset × Meal [High Fat 40g]				-1.04	-2.10, 0.01	0.053
Sleep offset × Meal [High Fibre]				-0.89	-2.08, 0.29	0.140
Sleep offset × Meal [High Protein]				-0.01	-1.08, 1.05	0.979
Sleep offset × Meal [MCM at home]				-1.31	-3.08, 0.47	0.151
Sleep offset × Meal [Medium fat/carb]				0.32	-0.63, 1.27	0.505
Random Effects						
ICC	0.45			0.45		
N	953 $_{username}$			953 $_{username}$		
	765 $_{family\_id}$			765 $_{family\_id}$		
Observations (*n* meals)	8395			8395		
Marginal R^2 / Conditional R^2	0.363 / 0.652			0.364 / 0.652		

[a] Sleep offset is expressed in hours as a deviation from midnight
MCM, metabolic challenge meal

Supplementary Table 7. Between-person sleep offset and onset and postprandial glucose

	Model without interaction			Model with interaction		
Predictors	*Estimates*	*CI*	*p*	*Estimates*	*CI*	*p*
(Intercept)	81.24	72.72, 89.76	<0.001	78.71	69.27, 88.16	<0.001
Sleep offset [a]	0.11	-0.30, 0.52	0.600	0.47	-0.24, 1.19	0.194
Sleep onset [a]	0.41	0.01, 0.81	0.047	0.42	0.01, 0.82	0.042
Meal [High Carb]	-15.92	-17.23, -14.61	<0.001	-9.88	-16.78, -2.99	0.005
Meal [High Fat 35g]	-42.42	-45.38, -39.46	<0.001	-39.47	-55.15, -23.80	<0.001
Meal [High Fat 40g]	-49.98	-51.39, -48.58	<0.001	-42.68	-50.21, -35.14	<0.001
Meal [High Fibre]	-21.20	-22.83, -19.57	<0.001	-14.84	-23.42, -6.26	0.001
Meal [High Protein]	-49.56	-51.11, -48.01	<0.001	-49.46	-57.24, -41.68	<0.001
Meal [MCM at home]	-36.55	-38.67, -34.44	<0.001	-27.55	-39.93, -15.16	<0.001
Meal [Medium fat/carb]	-27.90	-29.19, -26.61	<0.001	-30.11	-36.92, -23.30	<0.001
Sleep offset × Meal [High Carb]				-0.86	-1.82, 0.10	0.081
Sleep offset × Meal [High Fat 35g]				-0.42	-2.60, 1.77	0.709
Sleep offset × Meal [High Fat 40g]				-1.04	-2.10, 0.01	0.053
Sleep offset × Meal[High Fibre]				-0.90	-2.08, 0.29	0.139
Sleep offset × Meal [High Protein]				-0.02	-1.09, 1.05	0.975
Sleep offset × Meal [MCM at home]				-1.31	-3.09, 0.47	0.150
Sleep offset × Meal [Medium fat/carb]				0.32	-0.63, 1.27	0.505
Random Effects						
ICC	0.45			0.45		
N	953 $_{username}$			953 $_{username}$		
	765 $_{family\_id}$			765 $_{family\_id}$		
Observations	8395			8395		
Marginal R^2 / Conditional R^2	0.363 / 0.652			0.364 / 0.652		

[a] Sleep offset and onset are expressed in hours as a deviation from midnight
MCM, metabolic challenge meal

ESM Figure

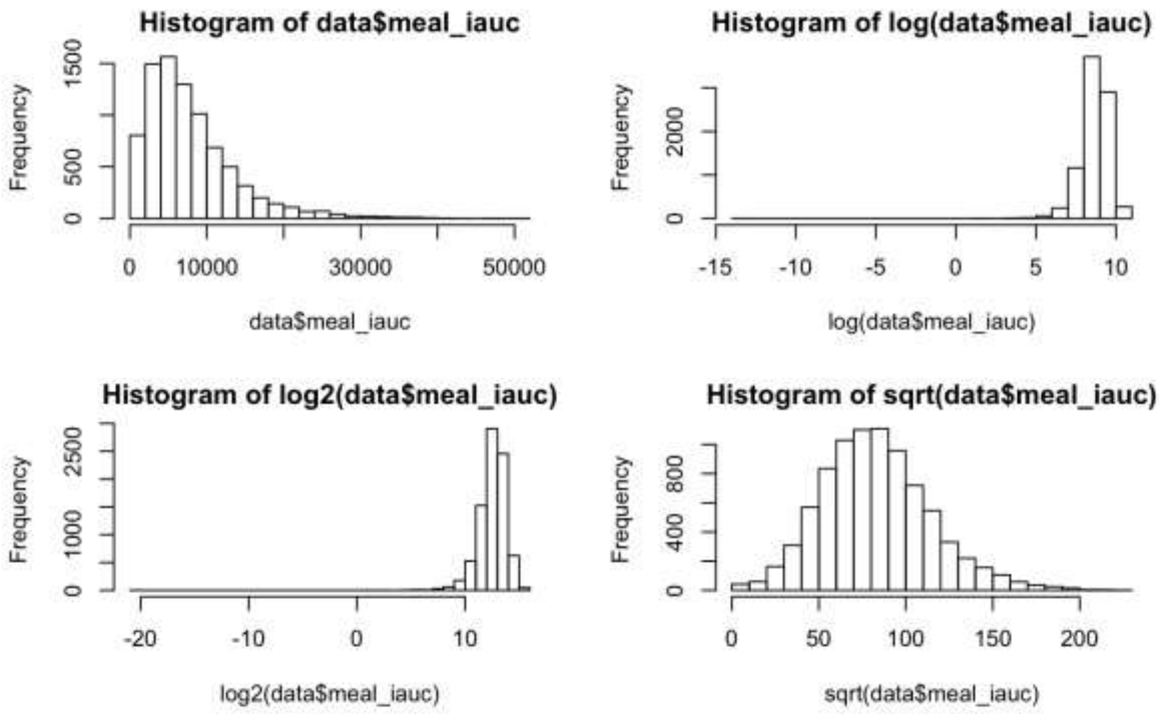

Supplementary Figure 1. Histograms of glucose$_{iAUC0\text{-}2h}$. From left to right: 1) Raw, 2) Natural log transformed, 3) Base 2 log transformed, 4) Square root transformed.
